# Association between plasma lipid levels during acute coronary syndrome and long-term malignancy risk. The ABC-4* study on heart disease

**DOI:** 10.1186/s12872-019-1092-5

**Published:** 2019-05-20

**Authors:** Giuseppe Berton, Rocco Cordiano, Fiorella Cavuto, Francesco Bagato, Heba Talat Mahmoud, Mattia Pasquinucci

**Affiliations:** 1 Department of Cardiology, Conegliano General Hospital, Via Brigata Bisagno, 31015 Conegliano, TV Italy; 2 ABC Study on Heart Disease Foundation ONLUS, Conegliano, Italy; 3 Department of Internal Medicine and Cardiology, Adria General Hospital, Adria, Italy; 4 Department of Cardiology, Bassano del Grappa General Hospital, Bassano del Grappa, Italy

**Keywords:** Acute coronary syndrome, Coronary artery disease, Neoplasia, Plasma lipids, Long-term follow-up, Competitive risks

## Abstract

**Background:**

Emerging evidence suggests that patients with coronary artery disease carry an increased risk of developing malignancy, with deleterious effects on long-term prognosis. Our aim was to ascertain whether baseline plasma lipid levels during acute coronary syndrome (ACS) are associated with malignancy in long-term.

**Methods:**

This study included 589 patients admitted with ACS to three centers and discharged alive. Plasma lipid levels were assessed on the first morning after admission. Patients were followed for 17 years or until death.

**Results:**

Five hundred seventy-one patients were free from malignancy at enrollment, of them 99 (17.3%) developed the disease during follow-up and 75 (13.1%) died due to it. Compared to patients without malignancy, those with malignancy showed lower plasma levels of total cholesterol (TC), low-density lipoprotein (LDL), and triglycerides (TG). The groups showed similar statin use rates at any time in follow-up. The incidence rate of neoplasia and neoplastic mortality was higher in patients with baseline TC or LDL values ≤ median; they showed 85 and 72% increased incidence rate of developing malignancy and 133 and 122% increased incidence rate of neoplastic death respectively. No differences were observed relative to HDL and TG levels. In survival analysis using Cox regression with parsimonious models, patients with baseline TC or LDL values > median, respectively, showed risks of 0.6(95% CI 0.4–0.9; *p =* 0.01) and 0.6(95%CI 0.4–0.9; *p =* 0.02) for malignancy onset, and 0.5(95% CI 0.3–0.8; *p =* 0.005) and 0.5(95% CI 0.3–0.8; *p =* 0.004) for neoplastic death. Similar results were obtained using competitive risk analysis with parsimonious models.

**Conclusions:**

This long-term prospective study of an unselected real-world patient sample showed that neoplasia onset and mortality are independently associated with low plasma TC and LDL levels at admission for ACS.

## Background

Cardiovascular disease (CVD) and cancer are the two main causes of mortality worldwide [1, 2]. Most investigations of prognosis following acute coronary syndrome (ACS) focus on cardiovascular events, and few examine long-term fatalities [3, 4]. However, emerging evidence suggests that patients affected by CVD, particularly coronary artery disease (CAD), carry an increased risk of cancer development, which has a deleterious effect on long-term prognosis [5, 6]. It is not yet understood which patients have this higher risk of cancer.

Several studies indicate that cancer risk and cancer-related mortality show an inverse relationship with plasma levels of total cholesterol (TC) and low-density lipoprotein (LDL) in the general population [7–13].To our knowledge, this relationship has not been investigated in patients with ACS.ACS is reportedly accompanied by substantial transient changes in the plasma lipid profile, including increases of plasma triglycerides (TG) and very low-density lipoproteins, and decreases of TC, high-density lipoprotein (HDL), and LDL levels [14, 15]. Notably, a 10% decrease in TC has been described [15], which is clinically significant and warrants measurement of serum lipids in patients with acute myocardial infarction (AMI) within the first hours after presentation.

In the present study, we investigated the possible association between plasma lipid profile during ACS (admission plasma lipid level) and the subsequent long-term cancer risk over 17 years of follow-up in an unselected sample of patients discharged alive after an index hospitalization with ACS.

## Methods

### Patients

The ABC Study on Heart Disease is an ongoing prospective investigation designed to represent, as closely as possible, an unbiased population of patients with ACS (www.abcstudy.foundation). The cohort includes Caucasian patients with definite ACS—including ST-elevation myocardial infarction (STEMI), non-ST elevation myocardial infarction(NSTEMI), or unstable angina—who were admitted to the intensive care units of the Adria, Bassano and Conegliano hospitals between June 1995 and January 1998. The original aim of the ABC study was to monitor these patients with regards to natural long-term history and to evaluate both non-fatal and fatal events, and causes of death. Another study aim was to investigate the prognostic value of multiple baseline clinical variables. Criteria for ACS diagnosis included the clinical presentation, electrocardiogram findings, and the presence of serum biochemical markers of necrosis [16, 17].

**Fig. 1:**
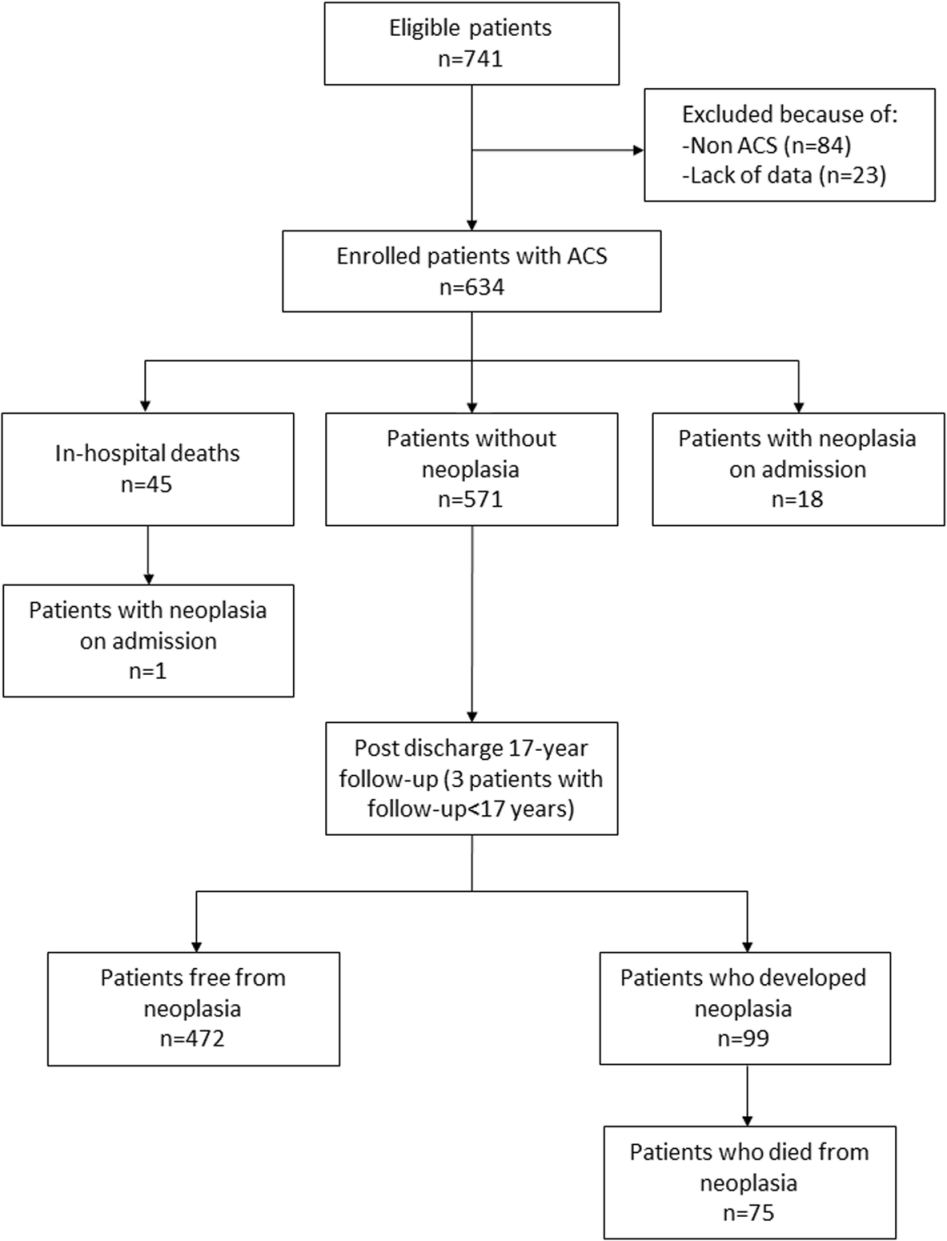
Flow Diagram of Patients’ Progress During Follow-Up. ACS = acute coronary syndrome

A total of 741 patients were considered eligible upon admission of whom 84 were excluded because they had diseases other than ACS, and 23 were excluded due to a lack of baseline data. Among the 634 enrolled patients with ACS, 45died during the index hospitalization; hence, the post-discharge follow-up study included 589 patients (Fig. 1). Malignant neoplasia had already been diagnosed in 19 patients at the time of enrollment, one of whom died during the index hospitalization. Each patient received an anonymous code, and no personal data or identifiers were included in the baseline or follow-up database. All enrolled patients gave their written informed consent, and the study was approved by each hospital ethics committee.

### Measurements and follow-up

At enrollment, thorough patient history was collected from medical records and patient interviews. All presently analyzed baseline clinical and laboratory data were obtained during the first 7 days of hospitalization in the intensive coronary care unit. ACS diagnosis criteria were the fulfillment of at least two of the following: central chest pain lasting > 30 min; typical changes in serum enzymes, including total creatine kinase (CK) and creatine kinase MB (CK-MB); and typical electro-cardiogram changes with pathological Q waves and/or localized ST-T changes in at least two contiguous leads [18]. Within 12 h after admission, a fasting venous blood sample was drawn for TC, LDL, HDL measurements. LDL concentrations were estimated using the modified Friedewald formula (MFF): LDL (mg/dL) = Non-HDL × 90% − TG × 10% [19]. In all three hospitals, plasma lipid measurement was performed using an enzymatic colorimetric method [20]. Details of the measured variables have been previously published [16, 17].

Each patient underwent a clinical check-up at 1, 3, 5, 7, 10, 12, 15, and 17 years after recruitment. At each recruitment hospital, two cardiologists were responsible for monitoring the cohort of patients throughout the follow-up. Data were obtained from scheduled examinations, public administrations, hospital records, family doctors, post-mortem examinations, and death certificates.

For the present study, the following data were recorded: presence of malignant neoplastic disease at the index admission; incidence of neoplastic disease and time of onset, i.e., the first documented clinical diagnosis of the disease; and time of death due to any cause. All patients were followed for 17 years or until the time of death. All data after enrollment were prospectively recorded following the protocol of the ABC Study on Heart Disease. By protocol, baseline data and follow-up data were recorded in two different data sheets. For the present analysis, the datasheets were merged after completion of 17 years of follow-up.

### Statistical analysis

The accrued variables were analyzed as continuous variables or proportions. Log transformations were applied to correct for positively skewed distributions, as appropriate. We analyzed measured variables using the unpaired Student’s t-test, and categorical variables using Pearson’s chi-square test. If a patient dropped out prior to 17 years of follow-up, her/his data were censored at that time. Survival curves were constructed using cumulative incidence as a function of neoplasia onset and neoplasia-related death [21]. We compared cumulative incidences using the Pepe and Mori Test [22] and incidence rates using Mantel-Haenszel estimates of the rate ratio. We analyzed the times from enrollment (i.e., admission for ACS) to the onset of neoplastic disease and to death using Cox proportional hazard regression analysis, as well as with competitive risk regression analysis using the Fine-Gray method [23]. Scaled Schoenfeld residuals were used to test the proportionality assumption with 95% confidence intervals (CI). All hazard ratios (HR) estimated in survival analysis were based on analysis of dichotomous variables, using the 50th percentile for continuous variables, and absence/presence of a feature for categorical variables. The same models were also assessed using the continuous baseline variables, and the strength of association expressed as Z values (the ratio of the HR and SE). The International System of Units is used throughout the text. Unless otherwise indicated, two-tailed *P* values of < 0.05 were considered significant. Statistical analyses were performed using STATA 14 (College Station, Texas, USA).

## Results

All enrolled patients completed the follow-up unless pre-empted by death—except three patients for whom survival time was censored before 17 years (two withdrew consent and one moved overseas). Among the589 patients who were discharged alive, 18patients had previously diagnosed malignancy at the time of enrollment and were excluded from the present analysis. Ninety-nine patients developed the disease during the follow-up (Fig. 1). Table 1 presents the patients’ baseline clinical characteristics according to the development of neoplasia during follow-up. The two groups did not differ in age at enrollment, history of hypertension or alcohol use. The prevalence of neoplasia was higher among males. Patients with neoplasia were more frequently smokers, and less frequently had diabetes or baseline signs of heart failure. Regarding humoral characteristics, patients with neoplasia had lower plasma levels of peak lactate dehydrogenase (LDH), TC, LDL, and TG. Plasma HDL levels did not differ between groups. The rate of using lipid-lowering treatment throughout follow-up did not significantly differ between non-neoplastic patients (47%) and neoplastic patients (43%)(chi^2^ = 2.9, *p* = 0.23).

**Table 1:** Baseline characteristics of patients with acute coronary syndrome by developing the neoplastic disease during follow-up

Variable	Overall sample (*n* = 571)	Non neoplastic (*n* = 472)	Neoplastic (*n* = 99)	*P* values
Median age. Years	67 (58–74)	67 (58–75)	67 (61–74)	0.71
Gender (female)	30	31	21	0.04
Education (above primary school)	26	26	26	0.93
Median body mass index. kg/m2	26 (24–28)	26(24–28)	25(24–29)	0.66
Smoking habit ^a^	67	65	80	0.003
Alcohol use	74	74	74	0.99
Hypertension	48	48	46	0.66
Diabetes mellitus	23	25	13	0.01
Median systolic blood pressure. mmHg	120 (110–130)	120 (110–130)	120 (110–130)	0.62
Median diastolic blood pressure. mmHg	80 (70–80)	76 (70–80)	80 (70–80)	0.10
Median heart rate. Beats/min	71(60–82)	72 (63–82)	70 (60–80)	0.07
non-ST elevation ACS	38	37	46	0.09
KIllip class > 1	66	36	22	0.008
LVEF (*n* = 500)	52 (45–60)	52 (45–60)	56 (46–61)	0.06
Hb (g/L)	137 (125–147)	137 (126–147)	137 (126–147)	0.88
Blood glucose level (mmol/L)	6.7(5.6–8.8)	6.8 (5.7–9.3)	6.2 (5.4–7.7)	0.05
Serum creatinine level (mmol/L)	0.08 (0.07–0.1)	0.08 (0.07–0.1)	0.08 (0.07–0.09)	0.06
CK-MB peak (U/L)^b^	103(43–205)	106(43–207)	78(34–186)	0.15
LDH peak (U/L)^b^	848(517–1380)	874(538–1418)	701(454–1200)	0.003
Serum lipids (mmol/L)^b^				
Total cholesterol	5.4(4.6–6.3)	5.5 (4.7–6.3)	5.2(4.4–6.2)	0.01
LDL cholesterol^c^	3.4(2.8–4.1)	3.5 (2.8–4.1)	3.3(2.6–4.0)	0.03
HDL cholesterol	1.1(1.0–1.3)	1.1 (1.0–1.3)	1.1(1.0–1.3)	0.73
Triglycerides	1.4(1.0–2.0)	1.5 (1.1–2.1)	1.3(0.9–1.9)	0.02

*ACS* Acute coronary syndrome, *CK-MB* Creatine kinase-MB isoenzyme, *HDL* High density lipoproteins, *LDH* Lactate dehydrogenase-1 isoenzyme, *LDL* Low density lipoproteins, *LVEF* Left ventricular ejection fraction, *Hb* Hemoglobin

The values are presented as medians and interquartile ranges or percentages

^a^Previous smokers and currently smoking patients

^b^*p* values were calculated on log-transformed data

^c^Calculated using modified Friedewald formula

For Hemoglobin: 1 g/L = 0.1 g/dl

For Glucose: 1 mmol/l = 18.01 mg/dl

For total cholesterol: LDL and HDL: 1 mmol/l = 38.66976 mg/dl

For Triglycerides: 1 mmol/l = 88.57396 mg/dl

Comparing patients who developed neoplasia to those who did not, there were no differences in the rate of revascularization; the rate of PCI was (17 and 21% respectively; chi^2^ = 0.66, *p* = 0.42) and of CABG was (17 and 20% respectively;chi^2^ = 0.34, *p* = 0.56).

The incidence rate of new malignancy throughout follow-up after ACS was approximately18 cases/1000 person-years. Unexpectedly, this incidence rate was markedly higher (23 cases/1000 person-years)among patients with baseline TC ≤ median value of 208 mg/dL, and the estimated rate ratio was significantly below 1 (Table 2). A similar rate ratio was observed for LDL. In contrast, the rate ratio was closer to 1and non-significant for HDL and TG.

**Table 2:** Incidence Rate of Neoplasia Onset, mortality and Comparison of Cumulative Incidence According to Lipid Levels

Variable	Person-years	Incidence rate/1000 person-years	Mantel-Haenszel estimates of rate ratio			Percent relative effect (%)	Pepe Mori cumulative incidence comparison	
			RR	X^2^	*p value*		X^2^	*p* value
Neoplasia onset after ACS (n = 99)	5544							
Total cholesterol								
≤ Median		23	0.54	8.8	0.003	85	7.4	0.006
> Median		13						
LDL cholesterol								
≤ Median		23	0.58	7.1	0.007	72	4.6	0.03
> Median		13						
HDL cholesterol								
≤ Median		17	1.10	0.2	0.63	−9	0.2	0.63
> Median		19						
Triglycerides								
≤ median		20	0.75	2.0	0.16	33	2.5	0.11
> median		15						
Neoplasia-related death after ACS (*n* = 75)	5877							
Total cholesterol								
≤ Median		18	0.43	12.1	0.0005	133	10.7	0.001
> Median		8						
LDL cholesterol								
≤ Median		18	0.45	11.4	0.0007	122	7.8	0.005
> Median		8						
HDL cholesterol								
≤ Median		11	1.33	1.6	0.8	−25	0.5	0.47
> Median		15						
Triglycerides								
≤ Median		15	0.68	2.7	0.09	47	1.6	0.20
> Median		11						

*ACS* Acute coronary syndrome, *HDL* High-density lipoproteins, *LDL* Low-density lipoproteins

At the end of follow-up, 75 (13.1%) patients had died due to neoplasia;(67 patients, died directly due to neoplasia,4 patients had concomitant non-cardiovascular adverse events likely contributing to death, and 4 patients had concomitant cardiovascular adverse events likely contributing to death). However, in the present analysis, we considered all the 75 patients died with malignancy as a single class of patients. The incidence rate approximated13 cases/1000 person-years. Among patients with TC ≤ median plasma values, the incidence rate was more than double of that observed among patients with TC > median value and the estimated rate ratio was highly significantly different (Table 2). Similar results were observed for LDL, while no significant differences were observed for HDL and TG (Table 2).

Overall, patients with TC or LDL baseline values > median value, had an increase of 85 and 72% in malignancy onset and 133 and 122% increase in neoplastic mortality, respectively, as compared to the patients with TC or LDL baseline values ≤median value.

**Fig. 2:**
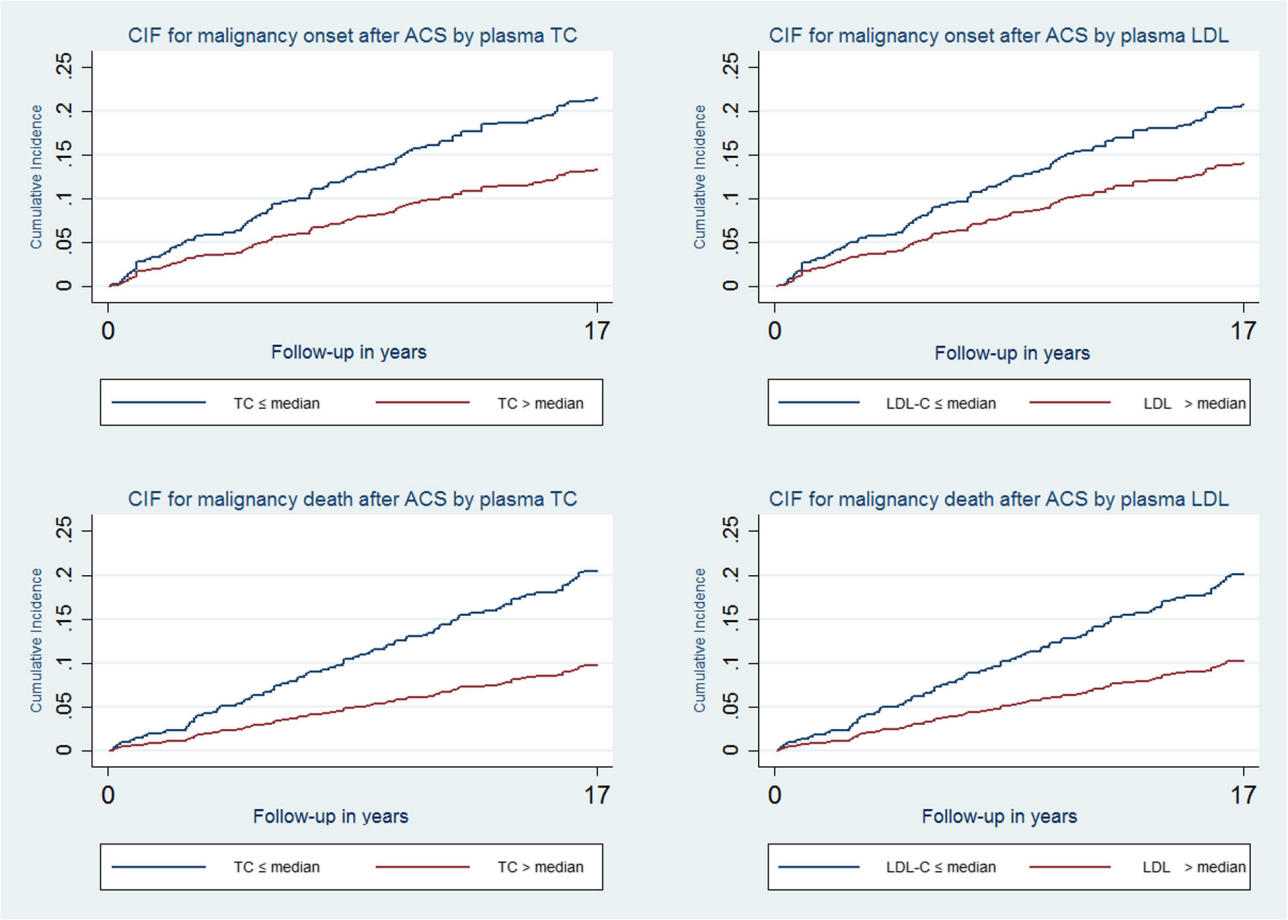
Cumulative Incidence Rate of Malignancy Onset and Neoplastic Death According to TC and LDL Values. ACS = acute coronary syndrome; CIF = denotes cumulative incidence function; LDL-C = low-density lipoprotein cholesterol; TC = total cholesterol

**Fig. 3:**
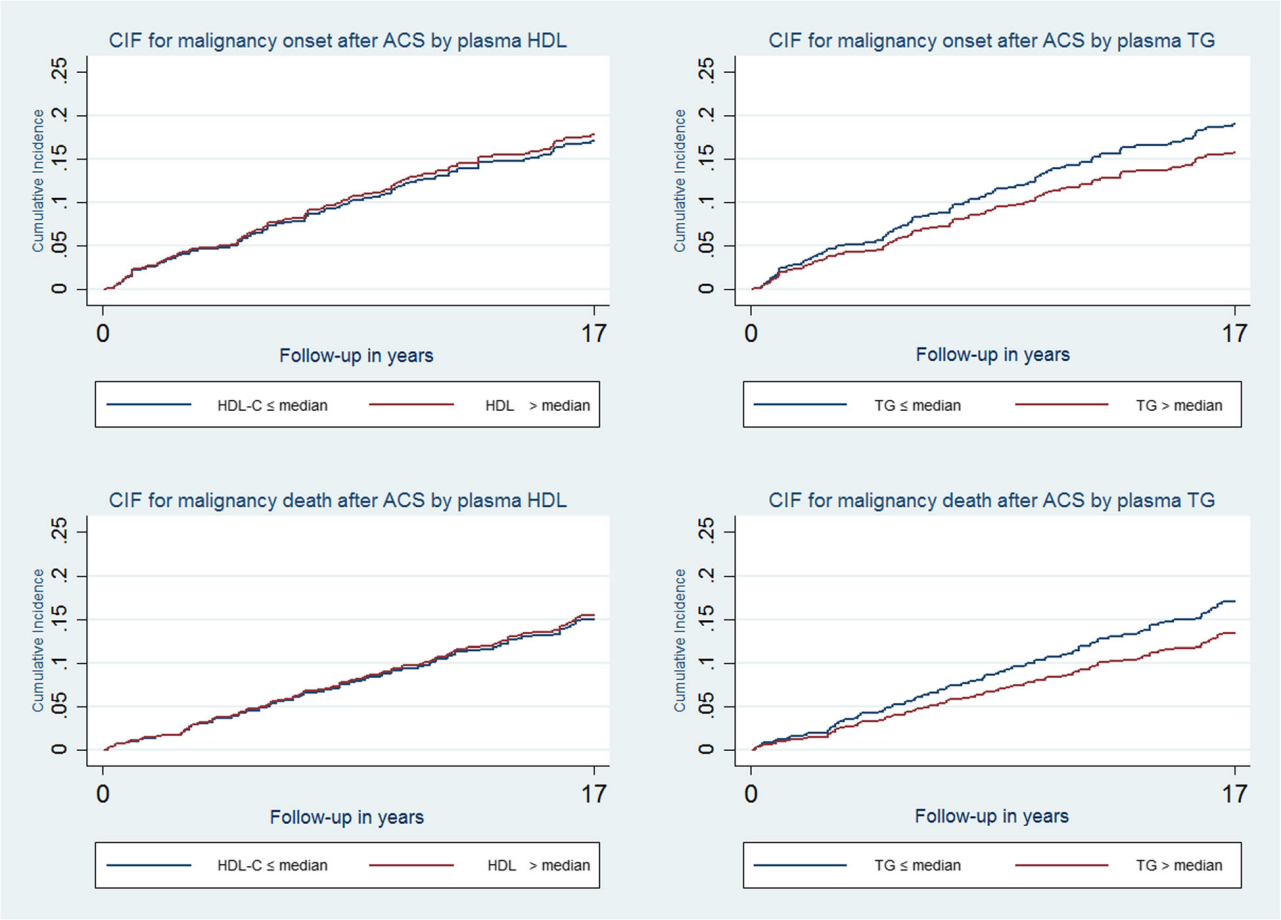
Cumulative Incidence Rate of Malignancy Onset and Neoplastic Death According to HDL And TG Values. ACS = acute coronary syndrome; CIF = cumulative incidence function; HDL-C = High density lipoprotein cholesterol; TG = Triglycerides

Figure 2 presents the cumulative incidence of malignancy onset and neoplastic death throughout the follow-up in patients with plasma TC and LDL values of > or ≤ median values, revealing significant differences between these groups (Table 2). There were no significant differences relative to HDL and TG (Fig. 3).

Univariable Cox survival analysis demonstrated that the hazard of malignancy onset and neoplastic mortality throughout follow-up after ACS were higher among patients with baseline TC or LDL values ≤ median values (Table 3). The proportional hazards assumption was verified for all variables concerning plasma lipid levels (*p* ≥ 0.10).

**Table 3:** Cox Regression and Competitive Risks Analysis for Neoplasia Onset and mortality after Acute Coronary Syndrome

Variable	Univariable analysis			Multivariable analysis					
				(fully adjusted model)^a^			(parsimonious model)		
	Hazard ratio (95% CI)	Z value	*p* value	Hazard ratio (95% CI)	Z value	*p* value	Hazard ratio (95% CI)	Z value	*p* value
Cox regression survival analysis									
Neoplasia onset (n = 99)									
Above median TC	0.6(0.4–0.8)	−2.9	0.003	0.6(0.4–0.9)	− 2.3	0.02	0.6(0.4–0.9)^b^	−2.6	0.01
Continuous TC		−3.6	< 0.0001		−3.0	0.003		−2.3^c^	0.002
Above median LDL-C	0.6(0.4–0.9)	− 2.6	0.009	0.6(0.4–0.9)	−2.0	0.04	0.6(0.4–0.9)^b^	−2.3	0.02
Continuous LDL-C		−3.2	0.001		−2.5	0.01		−2.8^c^	0.006
Above median HDL-C	1.1(0.7–1.6)	0.5	0.63	1.0(0.7–1.5)	−0.1	0.94	1.0(0.7–1.5)^c^	−0.1	0.89
Continuous HDL-C		0.03	0.74		−0.7	0.50		−0.3^c^	0.80
Above median TG	0.8(0.5–1.1)	−1.4	0.15	0.8(0.5–1.2)	−1.1	0.26	0.8(0.6–1.2)^c^	−0.9	0.35
Continuous TG		−3.0	0.003		− 2.1	0.03		−2.1^c^	0.04
Neoplasia-related death (n = 75)									
Above median TC	0.4(0.3–0.7)	−3.4	0.001	0.5(0.3–0.9)	−2.3	0.02	0.5(0.3–0.8)^b^	−2.8	0.005
Continuous TC		−4.3	< 0.001		−3.3	0.001		−3.7^c^	< 0.001
Above median LDL-C	0.4(0.3–0.7)	−3.2	0.001	0.5(0.3–0.9)	−2.4	0.02	0.5(0.3–0.8) ^b^	−2.9	0.004
Continuous LDL-C		−4.3	< 0.001		−3.3	0.001		−3.6^c^	< 0.001
Above median HDL-C	1.3(0.9–2.1)	1.3	0.20	1.1(0.7–1.8)	0.4	0.66	1.1(0.7–1.7)^c^	0.4	0.67
Continuous HDL-C		0.9	0.37		−0.1	0.91		0.21^c^	0.83
Above median TG	0.7(0.4–1.0)	−1.6	0.10	0.8(0.5–1.3)	−0.8	0.43	0.8(0.5–1.3)^c^	−0.9	0.37
Continuous TG		−3.0	0.003		−1.7	0.09		−1.8^c^	0.06
Competitive risks survival analysis									
Neoplasia onset (n = 99)									
Above median TC	0.6(0.4–0.9)	−2.6	0.01	0.6(0.4–0.9)	−2.4	0.02	0.6(0.4–0.9)^d^	−2.5	0.01
Continuous TC		−2.5	0.01		−2.6	0.01		−2.7	0.008
Above median LDL-C	0.7(0.4–0.9)	−2.1	0.04	0.6(0.4–0.9)	−2.0	0.04	0.7(0.4–0.9)^d^	−2.1	0.04
Continuous LDL-C		−2.3	0.02		−2.3	0.02		−2.4	0.02
Above median HDL-C	1.1(0.7–1.6)	0.2	0.82	1.0(0.7–1.5)	0.1	0.95	1.0(0.7–1.5)^d^	0.2	0.84
Continuous HDL-C		0.4	0.69		−0.1	0.93		0.3	0.79
Above median TG	0.8(0.5–1.2)	−1.0	0.30	0.8(0.5–1.3)	−0.9	0.36	0.8(0.5–1.2)^d^	−1.2	0.25
Continuous TG		−2.4	0.01		−2.4	0.02		−2.6	0.01
Neoplasia-related death (n = 75)									
Above median TC	0.5(0.3–0.8)	−3.1	0.002	0.5(0.3–0.9)	−2.5	0.01	0.5(0.3–0.8)^d^	−2.9	0.003
Continuous TC		−3.2	0.001		−2.8	0.006		−3.3^d^	0.001
Above median LDL-C	0.5(0.3–0.8)	−2.8	0.005	0.6(0.3–0.9)	−2.3	0.02	0.5(0.3–0.8)^d^	−2.7	0.007
Continuous LDL-C		−3.3	0.001		−2.7	0.007		−3.3^d^	0.001
Above median HDL-C	1.3(0.8–2.0)	1.0	0.32	1.2(0.8–1.9)	0.8	0.44	1.3(0.8–2.0)^d^	1.0	0.32
Continuous HDL-C		1.0	0.31		0.5	0.60		0.9^d^	0.35
Above median TG	0.7(0.5–1.2)	−1.3	0.19	0.8(0.5–1.3)	−0.8	0.41	0.7(0.5–1.1)^d^	−1.4	0.16
Continuous TG		−2.6	0.008		−2.1	0.04		−2.8^d^	0.006

*ACS* Acute coronary syndrome, *CI* Confidence interval, *HDL* High-density lipoproteins, *LDL* Low-density lipoproteins

^a^Adjusted for age, gender, BMI, smoking, diabetes mellitus, hypertension, in-hospital HF, Q-wave myocardial infarction, statin therapy, and hospital

^b^Adjusted for age, smoking, and Q-wave myocardial infarction

^c^Adjusted for age and smoking

^d^Adjusted for smoking and in-hospital HF

The higher hazard remained significant even after accounting for clinical confounders in the fully adjusted models and the parsimonious models (Table 3). Fully adjusted models included age, gender, body mass index, smoking habit, diabetes mellitus, hypertension, baseline in-hospital heart failure, Q-wave myocardial infarction, lipid-lowering treatment with statins, and hospital site. The proportional hazards assumption was also not violated for all lipids and for all other variables in the fully adjusted model (p ≥ 0.10), except for the presence of diabetes (*p* < 0.01).

The final survival analysis accounted for competitive risks (malignancy risk versus all other causes of death) and showed very similar results, both in univariable analysis and in the fully adjusted and parsimonious models (Table 3).The fully adjusted model showed that onset of malignancy was associated with smoking and HF at admission, the risks were 2.2(95% CI 1.2–4.1; *p* = 0.02) and 0.6(95% CI 0.3–1.0; *p* = 0.03) respectively, while the risks for neoplastic mortality were 2.5(95% CI 1.5–3.9; *p* = 0.00), 2.3(95% CI 1.3–4.3; *p* = 0.01) and 0.6(95% CI 0.4–1.0; *p* = 0.06) for age, smoking habits and HF at admission respectively. Possible interactions for TC and LDL were tested versus important baseline clinical variables(age, gender, the presence of hypertension, diabetes mellitus, smoking habit), revealing no interactions with any variables included in the fully adjusted model.

## Discussion

The results of this prospective study, virtually without drop-out patients, showed an independent higher risk of malignancy onset and mortality among patients with low TC and LDL values upon hospital admission for ACS. In the present analysis, all the patients were free of malignancy at enrollment. These results were consistent for both malignancy onset and mortality through 17 years of follow-up, and independent from important baseline clinical confounders, including age, gender, hypertension, diabetes mellitus, smoking habits, type of ACS, and heart failure. Furthermore, lipid-lowering treatment did not seem to influence the relationship of TC and LDL with cancer onset and mortality, with neoplasia incidence rates were similar between patients who did and did not receive statin medication during follow-up. Moreover, survival analysis controlling for lipid-lowering treatment during follow-up (both Cox regressions and competitive risks regressions) confirmed that the association was independent of the treatment.

Cancer and CVD are highly complex phenotypes and their concurrence is a controversial issue given the competing risks of mortality [24]. While inflammation and oxidative stress appear to be major unifying factors in the etiology and progression of both diseases, emerging evidence suggests that modifiable risk factors including unhealthy diet, sedentary lifestyle, obesity, and tobacco smoking are central to the pathogenesis of both diseases and are reflected in common genetic, cellular, and signaling mechanisms which have been thoroughly discussed [25–27].

Considering the dramatic prognostic severity of these clinical conditions, it is critical that we improve our understanding of this important biological overlap. Many observational cancer epidemiology studies showed that low cholesterol concentrations are associated with a significantly increased risk of total cancer and cancer-related mortality [7–13]although not all data support this relationship [28, 29].Regarding the possible explanations of this inverse association, authors suggest a direct causal link [30] while others discuss the possible effects of preclinical cancer [7].Other postulations include changes in cell membrane fluidity that lead to neoplastic transformation, reduced tumor immunogenicity secondary to membrane cholesterol loss, altered levels of fat-soluble antioxidants or vitamins transported in LDL particles, protective effects of LDL against lymphocyte activation, and virally induced cell transformation and genetic factors [30].

The relationship between plasma cholesterol concentration and mortality is complex. Although plasma concentration is positively correlated with CAD-related mortality, it shows a negative relationship with death from cancer. These two relationships could reflect causal mechanisms that are reversible by changes in plasma TC concentration. In this scenario, the benefits of lipid reduction for heart disease might be partly offset by increased cancer-related mortality [31].

In concordance with the medical knowledge, we found association between malignancy risk and other important variables as age and smoking, while interestingly the higher levels of cholesterol and LDL were consistently associated with lower malignancy risk.

Another important issue is how statin treatment during follow-up influences outcomes. The relationship between statin treatment and malignancy is controversial, as some studies report that statin-treated patients carry an increased risk of cancer in certain body segments [32-34], other studies report that statin treatment conveys a protective effect [35, 36] and several meta-analyses and observational studies have identified no association between statin use and overall cancer risk [37–43]. In a recent comprehensive review, the authors. Concluded that statin use seems to be safe in relation to cancer risk but that a preventive effect is not yet established [44].In our patient sample, statin treatment did not seem to have a significant influence on neoplastic onset or neoplastic death. The rates of neoplastic onset and death were similar between patients with and without treatment throughout follow-up. In the multivariable survival models, including those dealing with competitive risks assessment, statin treatment did not modify the association between plasma lipid levels and outcomes. Sub-analysis was performed among our patients who never received statin treatment throughout the entire study period, and the results support the hypothesis that the negative association between low admission plasma lipid levels (TC and LDL) is independent of treatment.

## Study limitations

A major limitation of the ABC study of ACS was that at the time of patient enrollment, percutaneous coronary angioplasty was not yet used to reopen coronary arteries in patients with STEMI. Thus, it remains uncertain whether the results might have been altered by early mechanical reperfusion. However, Cordero and his collogue reported recently that more than 86% of their patients have been subjected to revascularization post ACS and there were no differences in the revascularization rate among patients who did or didn’t develop neoplasia during the 7-year follow up [5]. Additionally, statin treatment was much less commonly used at the beginning of the study period (1995–1998), and steadily increased from the 1st to the 17th year of follow-up, in accordance with guideline revisions over the time period. However, our statistical analysis results suggested that lipid-lowering treatment did not influence the association of plasma lipid levels with cancer onset and mortality. Yet is to be considered that risk factors of occurrence of cancer vary by type of cancer, and it is of clinical relevance. However, this issue is beyond the scope of the present study, which aimed to assess the relationship between lipid and cancer incidence and death after ACS. One more limitation is that only baseline plasma lipid measurements were considered in the present study, while changes in lipid profile are to be expected through such a long time of follow up, mainly due to lifestyle and treatment changes. Nevertheless, the associations we observed seem to be clinically consistent, and the assessment of lipid profile at admission for ACS can be a sort key point in the patient’s life. Finally, since the patients in this study were all Caucasians, we cannot generalize the present findings to other populations and ethnic groups.

## Conclusions

This long-term prospective study of an unselected real-world patient sample showed that neoplasia onset and mortality are independently associated with low baseline plasma TC and LDL levels at admission for ACS.

## Abbreviations

ACS: Acute coronary syndrome
AMI: Acute myocardial infarction
CAD: Coronary artery disease
CI: Confidence intervals
CK: Creatine kinase
CK-MB: Creatine kinase MB
CVD: Cardiovascular disease
HDL: High-density lipoprotein
HR: Hazard ratios
LDH: Lactate dehydrogenase
LDL: Low-density lipoprotein
MFF: Modified Friedewald formula
NSTEMI: Non-ST elevation myocardial infarction
STEMI: ST-elevation myocardial infarction
TC: Total cholesterol
TG: Triglycerides

## References

[1] Benjamin EJ, Blaha MJ, Chiuve SE (2017). American Heart Association statistics committee and stroke statistics subcommittee. Heart disease and stroke statistics—2017 update a report from the American Heart Association. Circulation.

[2] Torre LA, Siegel RL, Ward EM, Jemal A (2016). Global cancer incidence and mortality rates and trends—an update. Cancer Epidemiol Biomark Prev.

[3] Fox KA, Carruthers KF, Dunbar DR (2010). Underestimated and under-recognized: the late consequences of acute coronary syndrome (GRACE UK-Belgian study). Eur Heart J.

[4] Berton G, Cordiano R, Palmieri R, Cavuto F, Pellegrinet M, Palatin P (2014). Prospective history of long-term mortality and modes of death in patients discharged after acute coronary syndrome: the ABC-2* study on acute coronary syndrome. Int J Cardiovasc Res.

[5] Cordero A, López-Palop R, Carrillo P (2018). Prevalence and post discharge incidence of malignancies in patients with acute coronary syndrome. Rev EspCardiol (Engl Ed).

[6] Iannaccone M, D'Ascenzo F, Vadalà P, et al. Prevalence and outcome of patients with cancer and acute coronary syndrome undergoing percutaneous coronary intervention: a BleeMACS substudy. Eur Heart J Acute Cardiovasc Care. 2018;7:631–8. 10.1177/2048872617706501 28593789

[7] Eichholzer M, Stähelin HB, Gutzwiller F, Lüdin E, Bernasconi F (2000). Association of low plasma cholesterol with mortality for cancer at various sites in men: 17-y follow-up of the prospective Basel study. Am J Clin Nutr.

[8] Palmier J, Lanzrath BJ (2012). Laboratory and biometric predictors of cancer-related mortality in an insured population. J Insur Med.

[9] Isles CG, Hole DJ, Gillis CR, Hawthorne VM, Lever AF (1989). Plasma cholesterol, coronary heart disease, and cancer in the Renfrew and Paisley survey. BMJ.

[10] Kitahara CM, Berrington de González A, Freedman ND (2011). Total cholesterol and cancer risk in a large prospective study in Korea. J Clin Oncol.

[11] Ahn J, Lim U, Weinstein SJ (2009). Prediagnostic total and high-density lipoprotein cholesterol and risk of cancer. Cancer Epidemiol Biomark Prev.

[12] Shor R, Wainstein J, Oz D (2007). Low Serum LDL Cholesterol Levels and the Risk ofFever, Sepsis, and Malignancy. Ann Clin Lab Sci.

[13] Benn M, Tybjærg-Hansen A, Stender S, Frikke-Schmidt R, Nordestgaard BG (2011). Low-density lipoprotein cholesterol and the risk of cancer: a mendelian randomization study. J Natl Cancer Inst.

[14] Khan HA, Alhomida AS, Sobki SH (2013). Lipid profile of patients with acute myocardial infarction and its correlation with systemic inflammation. Biomark Insights.

[15] Barth JH, Jackson BM, Farrin AJ (2010). SPACE ROCKET trial group change in serum lipids after acute coronary syndromes: secondary analysis of SPACE ROCKET study data and a comparative literature review. Clin Chem.

[16] Berton G, Citro T, Palmieri R, Petucco S, De Toni R, Palatini P (1997). Albumin excretion rate increases during acute myocardial infarction and strongly predicts early mortality. Circulation.

[17] Berton G, Cordiano R, Cavuto F, Giacomini G, De Toni R, Palatini P (2012). Predictors of ten-year event-free survival in patients with acute myocardial infarction (from the Adria, Bassano, Conegliano, and Padova hospitals [ABC] study on myocardial infarction). Am J Cardiol.

[18] Pasternak RC, Braunwald E, Sobel BE, Braunwald E (1997). Acute myocardial infarction. Heart disease.

[19] Chen Y, Zhang X, Pan B (2010). A modified formula for calculating low-density lipoprotein cholesterol values. Lipids Health Dis.

[20] Mizoguchi T, Edano T, Koshi T (2004). A method of direct measurement for the enzymatic determination of cholesteryl esters. J Lipid Res.

[21] Austin PC, Lee DS, Fine JP (2016). Introduction to the analysis of survival data in the presence of competing risks. Circulation.

[22] Pepe MS, Mori M (1993). Kaplan-Meier, marginal or conditional probability curves in summarizing competing risks failure time data?. Stat Med.

[23] Fine JP, Gray RJ (1999). A proportional hazards model for the subdistribution of a competing risk. J Am Stat Assoc.

[24] Bayliss EA, Reifler LM, Zeng C, McQuillan DB, Ellis JL, Steiner JF (2014). Competing risks of cancer mortality and cardiovascular events in individuals with multimorbidity. J Comorb.

[25] Masoudkabir F, Sarrafzadegan N, Gotay C, et al. Cardiovascular disease and cancer: Evidence for shared disease pathways and pharmacologic prevention. Atherosclerosis. 2017;263:343–51. 10.1016/j.atherosclerosis.2017.06.001 PMC6207942 28624099

[26] Berton G, Cordiano R, Cavuto F, Bagato F, Segafredo B, Pasquinucci M (2018). Neoplastic disease after acute coronary syndrome: incidence, duration, and features: the ABC-4* Study on Heart Disease. J Cardiovasc Med.

[27] Koene RJ, Prizment AE, Blaes A, Konety SH (2016). Shared risk factors in cardiovascular disease and cancer. Circulation..

[28] Pursnani A, Massaro JM, D'Agostino RB, O'Donnell CJ, Hoffmann U (2017). Guideline-based statin eligibility, Cancer events, and noncardiovascular mortality in the Framingham heart study. J Clin Oncol.

[29] Iso H, Ikeda A, Inoue M, Sato S, Tsugane S, JPHC Study Group (2009). Serum cholesterol levels in relation to the incidence of cancer: the JPHC study cohorts. Int J Cancer.

[30] Meilahn EN, Ferrell RE (1993). ‘Naturally occurring’ low blood cholesterol and excess mortality. Coron Artery Dis.

[31] Kritchevsky SB, Kritchevsky D (1992). Serum cholesterol and cancer risk: an epidemiologic perspective. Annu Rev Nutr.

[32] Alsheikh-Ali AA, Maddukuri PV, Han H, Karas RH (2007). Effect of the magnitude of lipid lowering on risk of elevated liver enzymes, rhabdomyolysis, and cancer: insights from large randomized statin trials. J Am Coll Cardiol.

[33] Goldstein MR, Mascitelli L, Pezzetta F (2008). Do statins prevent or promote cancer?. Curr Oncol..

[34] Ford I, Murray H, Packard CJ, Shepherd J, Macfarlane PW, Cobbe SM, on behalf of the west of Scotland coronary prevention study group (2007). Long-term follow-up of the west of Scotland coronary prevention study. N Engl J Med.

[35] Neil A, Cooper J, Betteridge J (2008). Reductions in all-cause, cancer, and coronary mortality in statin-treated patients with heterozygous familial hypercholesterolemia: a prospective registry study. Eur Heart J.

[36] Friis S, Poulsen AH, Johnsen SP (2005). Cancer risk among statin users: a population-based cohort study. Int J Cancer.

[37] Bjerre LM, LeLorier J (2001). Do statins cause cancer? A meta-analysis of large randomized clinical trials. Am J Med.

[38] Kaye JA, Jick H (2004). Statin use, cancer risk in the general practice research database. Br J Cancer.

[39] Dale KM, Coleman CI, Henyan NN, Kluger J, White CM (2006). Statins and cancer risk: a meta-analysis. JAMA.

[40] Kuoppala J, Lamminpaa A, Pukkala E (2008). Statins and cancer: a systematic review and meta-analysis. Eur J Cancer.

[41] Alsheikh-Ali AA, Trikalinos TA, Kent DM, Karas RH (2008). Statins, low-density lipoprotein cholesterol, and risk of cancer. J Am Coll Cardiol.

[42] Browning DR, Martin RM (2007). Statins and risk of cancer: a systematic review and meta analysis. Int J Cancer.

[43] Bonovas S, Filioussi K, Tsavaris N, Sitaras NM (2006). Statins and Cancer risk: a literature-based Meta-analysis and Meta-regression analysis of 35 randomized controlled trials. J Clin Oncol.

[44] Boudreau DM, Yu O, Johnson J (2010). Statin use and cancer risk: a comprehensive review. Expert Opin Drug Saf.

