# Correction to: Association between plasma lipid levels during acute coronary syndrome and long-term malignancy risk. The ABC-4* study on heart disease

**DOI:** 10.1186/s12872-021-01974-y

**Published:** 2021-06-14

**Authors:** Giuseppe Berton, Rocco Cordiano, Fiorella Cavuto, Francesco Bagato, Heba Talat Mahmoud, Mattia Pasquinucci

**Affiliations:** 1 Department of Cardiology, Conegliano General Hospital, Via Brigata Bisagno, 31015 Conegliano, TV Italy; 2 ABC Study on Heart Disease Foundation ONLUS, Conegliano, Italy; 3 Department of Internal Medicine and Cardiology, Adria General Hospital, Adria, Italy; 4 Department of Cardiology, Bassano del Grappa General Hospital, Bassano del Grappa, Italy

## Correction to: BMC Cardiovascular Disorders (2019) 19:119 10.1186/s12872-019-1092-5

Following publication of the original article [1], an error was identified in the article. The final version has incorrectly contained the proofs watermarking.

The original article has been corrected as well.
